# Determining the 'point of change' in the patient's history - The delta rule

**DOI:** 10.5116/ijme.554f.858b

**Published:** 2015-05-24

**Authors:** Ami Schattner

**Affiliations:** Hebrew University Hadassah Medical School, Jerusalem, Israel

**Keywords:** Medical encounter, Medical history, Communication, Patient-centered care

## Introduction

Time in clinical encounters is notoriously short, and time constraints are a major acknowledged problem in almost every setting, with a worldwide prevalence.^1^^,^^2^ Arguably, the encounters most vulnerable to time shortage take place when clinicians in training (medical students, residents) meet a highly varied mix of patients whom they see for the first time in the crowded, busy atmosphere of the emergency department (ED). Their task is often further confounded by the patients and families who tend to flood and overwhelm the provider with endless, poorly-arranged and badly-presented history details, contributing to information chaos.^3^ Problems in getting a coherent medical history within a given time limit apply to other settings as well, such as the department of medicine. We suggest applying "The ∆ Rule" as a potential tool to help providers to quickly understand the patient's story and present initial data on its acceptability and efficacy.

## Methods

The technique is simple to teach and apply: immediately after the chief complaint is clarified, the provider should give first priority to determine the exact point in time when the patient's condition started to change with new symptoms culminating in the current presentation (the "∆ Point", delta signifying 'change' in mathematics). This is usually quite easily accomplished, yielding a point in time (e.g. "2 hrs after midnight" or "3 weeks ago"). At this stage, enquiry after the nature of the phenomena can be disregarded, simplifying our quest, since all we want to know at this stage is "When?". This approach establishes the timing as the hinge point, but does not replace elicitation of all dimensions of the chief complaint. Once determined - the "time frame" of the story can be clearly visualized. Then, the provider needs to think of, and 'cover' just two directions - backwards and forward, in that chronological order. First, going backwards will obtain an idea of what the patient's life was like before: i.e. the "background" characteristics preceding the 'present illness' (PI). Only then, when the time frame is set and the pre-illness profile (pattern) is established, can the PI be fully appreciated by moving forward to fully understand the immediate preceding circumstances (if any) and the step by step evolving signs and symptoms in full detail and chronological order. A short review of risk factors (family history, occupational / travel / pet exposures, iatrogenic factors, habits, etc.) concludes the history, and the examination can begin. The technique can benefit medical students, residents and providers in primary care, but attending physicians and consultants may find it as useful. When the patient cannot cooperate due to pain, distress or confusion - the same technique can be applied to family members or a care giver.

### Current evidence

"The ∆ Rule" is an efficient method of getting the essential data right and I have been using it to advantage for several years, before teaching it to students and collecting the current data. In my experience, it encourages patients to think about their illness clearly and describe it as it developed. Thus, the provider is able to quickly establish a lucid frame of the illness narrative with all of its components which stand out compared to the patient's past. On the patient's side, the patient will sense that his or her illness narrative was better understood, with the potential for improved satisfaction and trust, better patient-physician relationship and possibly improved health outcomes.^4^ Seven groups of medical students in their final years (n=61) were given "The ∆ Rule" in the middle of their 13-week department of medicine clerkship as a short (~40 min), small-group tutorial by the author, immediately followed by a single demonstration on an arbitrarily-selected patient who was recently admitted (~20 min). A questionnaire was distributed at the end of their program. The short, single instruction period notwithstanding, 93% (57/61) used "The ∆ Rule" regularly since it was presented. Most users also considered it to be 'highly useful' (50/57, 88%) - best on a visual analogue scale of 5 options (the remainder said it was 'useful'). Getting the history is highly context-dependent and variable between patients and between students. Nevertheless, when ten students (10/57, 17.5%) timed themselves twice during history-taking, just before the instruction and two weeks after, marked differences were found. The procedure took 39.2 ± 3.8 min before vs. 27.5 ± 3.0 min after "The ∆ Rule" was demonstrated and applied. The quality and clarity of their written admission notes also improved changing from 71.5 ± 6.7 to 87.5 ± 6.4 after the intervention (marks out of 100, graded by the author). Thus, a time gain of 11.7 min and an improved quality of 16.0 points was suggested by these initial data. Limitations of the study include the small number of participants in the timing experiment and potential bias in the grading of the students' notes.

## Conclusion

The patient's history remains the single most powerful element enabling correct diagnosis of most patients in either ambulatory^5^ or hospital^6^ settings. Any method of getting it in a fast and accurate fashion should prove highly useful. "The ∆ Rule" is suggested as an improved way of obtaining the essence of the patient's history in a clear, properly-sequenced and time-efficient way. It needs to be confirmed by further, more rigorous evaluation in the future. Evaluation of the patient's point of view is also mandatory: satisfaction would also support its adoption. Until then, it can already be used as a 'user-friendly' way of capturing the gist of the patient's story^7^which is time-frugal and easy to adopt and apply. 

### Conflict of Interest

The author declares that there are no conflicts of interest.
